# Large-area silicon photonic crystal supporting bound states in the continuum and optical sensing formed by nanoimprint lithography†

**DOI:** 10.1039/d3na00001j

**Published:** 2023-01-25

**Authors:** Huijuan Zhao, Xinyi Cao, Qiao Dong, Chunyuan Song, Lianhui Wang, Li Gao

**Affiliations:** a Nanjing University of Posts and Telecommunications, School of Materials Science and Engineering, State Key Laboratory for Organic Electronics and Information Displays China iamlgao@njupt.edu.cn iamlhwang@njupt.edu.cn

## Abstract

Optical bound states in the continuum (BIC) are found in various dielectric, plasmonic and hybrid photonic systems. The localized BIC modes and quasi-BIC resonances can result in a large near-field enhancement and a high-quality factor with low optical loss. They represent a very promising class of ultrasensitive nanophotonic sensors. Usually, such quasi-BIC resonances can be carefully designed and realized in the photonic crystal that is precisely sculptured by electron beam lithography or interference lithography. Here, we report quasi-BIC resonances in large-area silicon photonic crystal slabs formed by soft nanoimprinting lithography and reactive ion etching. Such quasi-BIC resonances are extremely tolerant to fabrication imperfections while the optical characterization can be performed over macroscopic area by simple transmission measurements. By introducing lateral and vertical dimension changes during the etching process, the quasi-BIC resonance can be tuned over a wide range with the highest experimental quality factor of 136. We observe an ultra-high sensitivity of 1703 nm per RIU and a figure-of-merit of 65.5 for refractive index sensing. A good spectral shift is observed for detecting glucose solution concentration changes and adsorption of monolayer silane molecules. Our approach involves low-cost fabrication and easy characterization process for large-area quasi-BIC devices, which might enable future realistic optical sensing applications.

## Abstract 2

The quasi-BIC modes are formed in silicon photonic crystal slabs by controlling both the lateral and vertical etched geometries. A high *Q* factor of 136, sensitivity of 1703 nm per RIU and figure-of-merit of 65.5 are observed in the optical sensor experiments.

## Graphical abstract

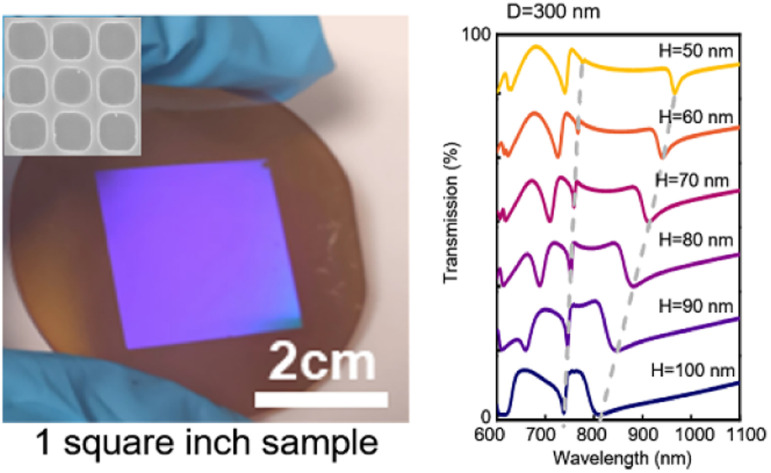

## Introduction

In the past, plasmonic devices have been the key players in high-performance optical sensors^1^ as the collective oscillation of surface plasmons in metallic nanostructures is highly sensitive to the minute changes of local environmental perturbation.^2–6^ The plasmonic resonance can be carefully designed to obtain Fano resonance, where the interference of a continuum state and a discrete state can yield an asymmetrical spectral line shape with relatively high-quality resonance. Therefore, the nanoplasmonic device can achieve a high refractive index sensitivity (*S*, defined as *S* = Δ*λ*/Δ*n*) over 1000 nm per RIU and a single molecular detection limit. Such a formation of high-performance plasmonic resonance always requires precise control over the fabrication process and advanced optical characterization. In real application scenarios, plasmonic resonances are usually associated with large optical losses and broad experimental resonance line shapes. The resonance quality factor (*Q*, defined as *Q* = *f*_r_/Δ*f*, where *f*_r_ is the resonance frequency and Δ*f* is the bandwidth) and figure of merit (FOM, defined as FOM = *S*/FWHM) are relatively low, which ultimately affect the sensor performance and the detection limit.^7^

Recently, the concept of bound states in the continuum (BIC) has been examined using theoretical models for experimental realization in various fields. A BIC can be considered as a non-radiating resonant mode that cannot couple with the radiating channels propagating outside the system, thus, they are also termed the “dark mode”. For the first time, Dong *et al.* mapped out and proved the strong near-field localization of the true BIC resonance on arrays of silicon nanoantennas *via* electron energy loss spectroscopy with a sub-1 nm electron beam.^8^ The most widely investigated BIC modes are the symmetry-protected BIC and the accidental (Friedrich–Wintgen BIC).^9–11^ The symmetry-protected BIC originates from the forbidden coupling between the eigenmodes of resonators and the external propagating modes, resulting in an infinite *Q*-factor with a vanishing spectral linewidth. Such BIC appears at the centre of the Brillouin zone (the *Γ* point) in photonic systems. They are robust to geometric changes as long as the symmetry is preserved. However, once the structural asymmetry is introduced, the symmetry-protected BIC will turn into a quasi-BIC mode with a finite *Q*-factor and linewidth. Such emissive quasi-BIC resonances can become visible and they are able to enhance localized light emission *via* the Purcell effect by at least 60 times as compared to the unpatterned silicon.^8^ The structural asymmetry and the *Q*-factor of resonances are found to be well described by a characteristic inverse-square law, thus allowing a fine-tuning of desirable high *Q*-resonances.^12–16^ The accidental BIC occurs when two discrete resonant states interfere when the asymmetric Fano-resonance is present. When the system parameters are tuned and lead to complete destructive interference of two resonant states, the Fano resonance collapse and the linewidth of the BIC mode disappears. Such BICs are at the off-*Γ* points and can be obtained by tuning the geometric parameters.

The dielectric photonic crystal (PhC) slab has periodic modulation of the refractive index in one-, two-, or three-dimensions. They can support BIC resonance by either symmetry-protected (*Γ* point) or accidental BIC (off-*Γ* point) that allows high-performance lasing^17,18^ and sensing^19–21^ applications. In such a system, the BIC modes are based on antisymmetric Bloch surface waves tightly confined in the vertical direction that cannot couple with the continuous spectrum of radiating waves. Quasi-BIC based on symmetry-protected BIC occurs if fabrication imperfections and the finite extent of the structure break the ideality of the system, which does not depend on the structural geometry. Recently, advanced designs of Si nanoantennas through gradient descent algorithm can support two partially overlapping quasi-BIC modes for sharp spectral edges at red wavelengths and suppress high-order modes at blue/green wavelengths, which results in Schrödinger's red pixels with ∼80% reflectance.^22^ The occurrence of the accidental BIC or its quasi-BIC depends on the destructive interference of resonant modes, which is determined by the actual structural parameters. Moreover, quasi-BIC resonances usually require index-matched surrounding and angled excitation.^23–26^ In this work, we investigated the BIC modes in a large-area silicon PhC slab formed by soft nanoimprint lithography (SNL) and reactive ion etching (RIE). Nanohole and nanopillar patterns are chosen because such BIC formation can be induced by both the symmetry-protected mode and accidental mode; thus, the BIC resonance characteristics can be tuned by both symmetry-breaking factors and geometric parameters. We demonstrate that the quasi-BIC mode formed in such a system is primarily due to accidental BIC, which is sensitive to the lateral etching dimension and vertical etching depth. In other words, the dimensional change of PhC can actually tune the BIC resonance position and the *Q*-factor. These large-scale BIC resonances are extremely tolerant to the fabrication imperfections introduced in the processing, yielding an experimental *Q*-factor comparable with those processed by electron beam lithography. Our silicon PhC slab can be made centimetres large and the optical characterization does not require any microscopic alignment. We also demonstrate fast and easy sensing experiments of the refractive index, solution concentration, and monolayer molecule adsorption.

## Results and discussion

**Fig. 1:**
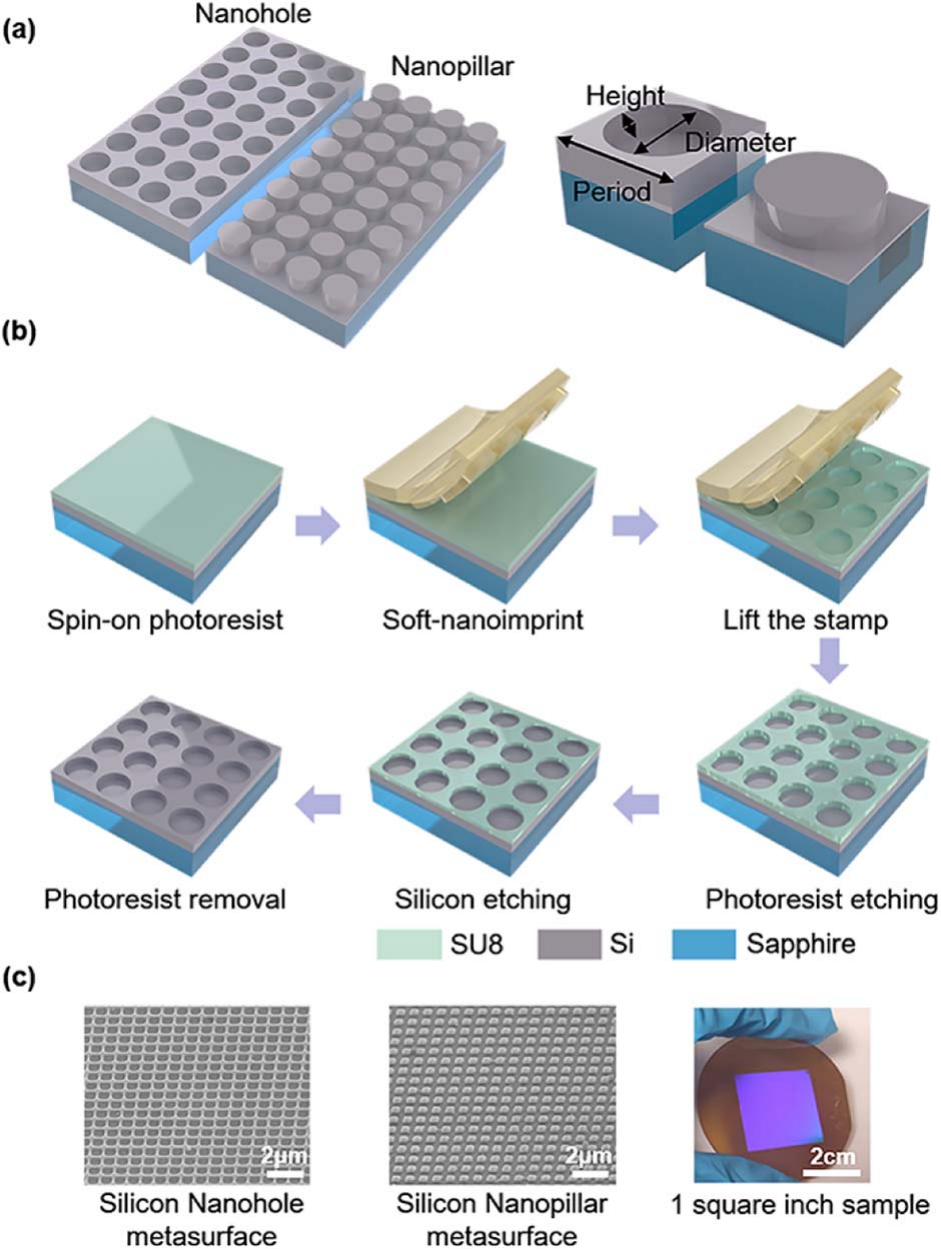
Illustration of the silicon PhC structures. (a) The periodic nanohole and nanopillar silicon photonic crystal nanostructure formed by the soft nanoimprint lithography on a sapphire substrate, the period is fixed while the nanohole (nanopillar) diameter *D* and etched depth *H* is varied. (b) The process flow of soft nanoimprint lithography and reactive ion etching. (c) Scanning electron microscope images and photographs of representative samples prepared in the experiment.

In order to obtain high *Q* quasi-BIC resonance in symmetry-protected conditions, asymmetry is introduced through various structure truncation, distortion, and rotation, such as nanobars, nano ellipsoids, and crescents.^27–29^ They can only be obtained by advanced nanofabrication techniques, such as electron beam lithography with a small device area. Although an ultrahigh *Q* factor over a few thousand can be obtained in theoretical quasi-BIC dielectric resonators, a slight geometrical broadening effect introduced in electron beam lithography (EBL) can result in a typical experimental *Q* factor slightly greater than 100.^28^ Such discrepancy is associated with the unavoidable material and structural imperfections, and defects induced during the fabrication. In addition, the optical measurement of small-area devices requires a sophisticated measurement system with precise alignment, while large-area devices are convenient to handle fast measurements. For future large-scale photonic device applications associated with microfluidic multi-channel sensor measurements, high-throughput and low-cost techniques, such as photolithography and nanoimprint lithography are most promising. In our previous work, we proposed a large-area and low-cost method to fabricate a large-area silicon-on-sapphire photonic crystal through a combination of soft nanoimprint SNL and RIE.^30^ Here, we apply this approach to form a large area silicon PhC slab and investigate the quasi-BIC resonance based on accidental BIC modes in such a photonic system. The simple periodic nanohole and nanopillar Si PhC slabs explored are illustrated in Fig. 1a. We used a plasma-enhanced chemical vapor deposition (PECVD) to form a 100 nm thick amorphous silicon on a transparent sapphire substrate. By coating a thin layer of the SU8 photoresist, a primary stamp could transfer a large-area periodic nanohole or nanopillar pattern into the SU8 photoresist with a thickness contrast. During the RIE process, the first step of O_2_ etching could etch away the thin photoresist residue at the bottom, and this etching step can vary the lateral dimension of the PhC nanohole or nanopillar. The subsequent CHF_3_/SF_6_ etching will etch the underlying silicon slab with different depths with careful control of the etching conditions and time. Due to the facility limitation, we did not perform a cross-sectional examination as done for the multi-layer devices,^31^ but this single silicon layer thickness can be well controlled and estimated from the etching time. The last step was the O_2_ mask removal process (Fig. 1b). At a fixed 400 nm period, the nanohole diameter is varied from 310–370 nm and the nanopillar diameter is varied from 260–210 nm. The complete sample area is 6.25 cm^2^. The structure is generally uniform over the whole surface for the same sample under identical etching conditions. However, we could observe imperfect unit cell shapes (neither perfectly round nor square shaped) and structural variations with dimension changes of a few nanometres at different regions (Fig. 1c), which are unavoidable and characteristic results of SNL and RIE techniques.

**Fig. 2:**
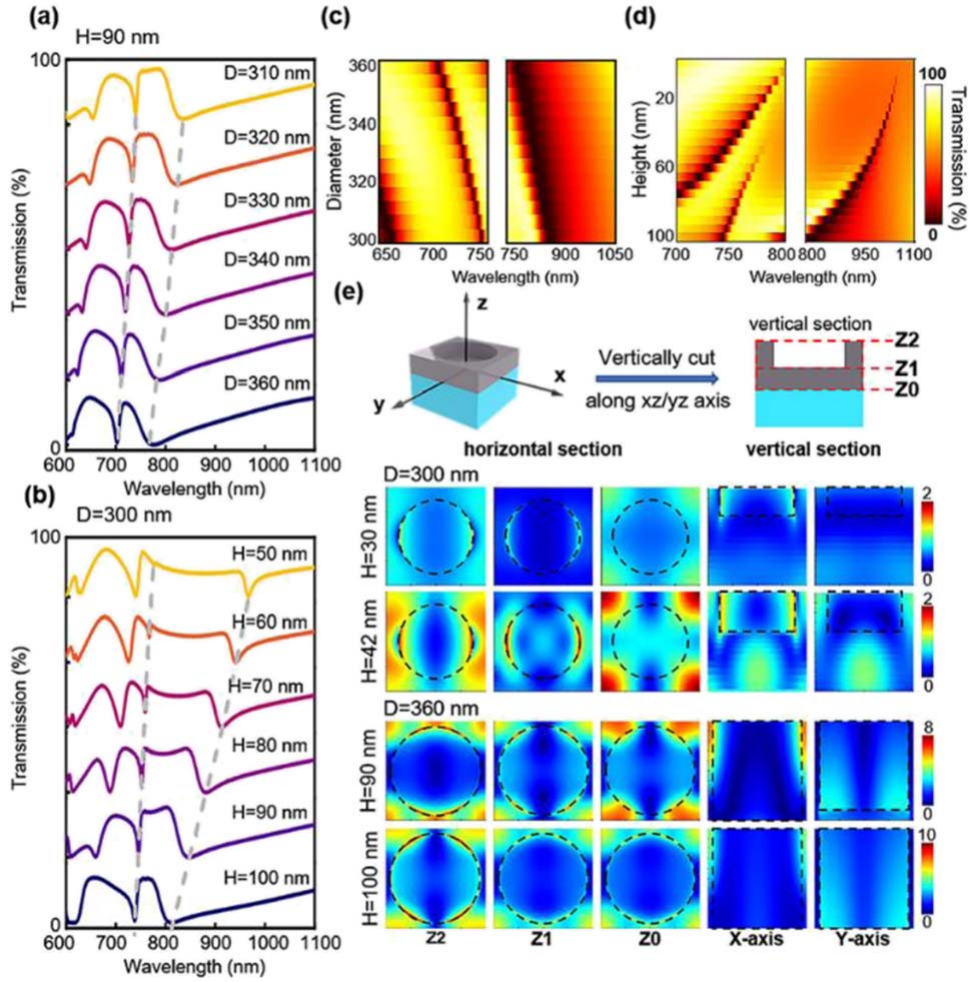
(a) Simulated transmission spectra of nanoholes while fixing the etching depth at *H* = 90 nm, diameter varies from 310 to 360 nm. (b) Simulated transmission spectra of nanoholes while fixing the diameter of 300 nm, the etching depth varies from 50 nm to 100 nm. (c) Simulated transmission spectra of nanoholes with different diameters. (d) Simulated transmission spectrum of nanoholes with different etching depths. (e) The electric field arrow map superimposed with the amplitude distribution of mode 1 from various views. Top: schematic diagram of a single lattice. Middle: fixing diameter at 300 nm, etching depth *H* = 30 and 42 nm. Bottom: fixing diameter at 360 nm, etching depth *H* = 90 and 100 nm.

**Fig. 3:**
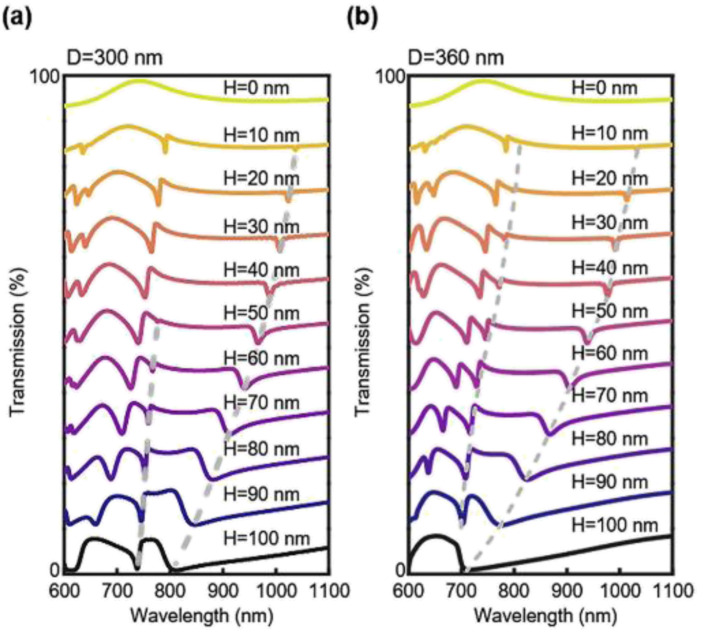
(a) Simulated transmission spectra of nanoholes with the diameter of 300 nm while etching depth increases from 0 to 100 nm. (b) Simulated transmission spectrum of nanoholes with the diameter of 360 nm while etching depth increases from 0 to 100 nm.

Before the device fabrication, we have used finite-domain-time-domain (FDTD) simulation to investigate the accidental BIC modes in the system. Simulations have been performed with a plane wave under normal incidence with the wavelength from 400–1400 nm. The periodic boundary condition has been applied along the *X* and *Y* directions while the perfectly matching layer is set along the *Z* direction. We have varied the lateral diameter of the nanoholes from 310 nm to 360 nm, while fixing the etching depth (*H*) to 90 nm. As shown in Fig. 2a, there are two main resonance peaks (at 700–740 nm, named mode 1; at 770–840 nm, named mode 2) corresponding quasi-BIC with a *Q* factor in the range of 6–346. The simulation results for the nanohole diameter variations are shown in Fig. 2b, demonstrating that the mode position can shift to a shorter wavelength with a larger hole diameter. While the resonance position is tuned by the hole diameter, the shape or the *Q* factor of the resonance does not change much. In the second scenario, we have fixed the hole diameter to 300 nm and varied the silicon etching depth from 0 nm to 100 nm. The spectral resonances from *H* = 50 nm to *H* = 100 nm are plotted in Fig. 2c. The complete spectral shifts are presented in Fig. 2d and 3a. To our surprise, the vertical silicon thickness variation actually plays a more prominent role in inducing a transition of a true BIC to quasi-BIC, which is observed at 42 nm, in the case of mode 1 resonance. BIC explored in a bilayer photonic crystal reveals that broken mirror-flip symmetry in the *Z* direction can give rise to high *Q* BIC states, which may suggest that our vertical thickness variation *H* of the nanoholes can impact the accidental BIC modes.^32^ For *D* = 300 nm, the quasi-BIC formation first occurrs at *H* = 42 nm and more quasi-BIC resonances appears when *H* is greater than 42 nm. In both cases, the electrical near-field at the interfaces is enhanced significantly due to the generation of a local leaky resonant state. For the etching depths smaller than 42 nm, for example, *H* = 30 nm, the BIC mode is destructively interferenced and nonradiating, thus no particular field localization and enhancement are observed at the interfaces (Fig. 2e). When etching depth is greater than 42 nm, the electric fields are greatly enhanced at planes *Z*_2_ and *Z*_1_, which is important for sensor applications at the device surface. In addition, the shape of the mode 1 resonance is greatly affected by the vertical etching depth, and the *Q* factor decreases to 82, as the etching depth is further increased to 100 nm. As *H* increases from 10 nm to 100 nm, the *Q* factor of mode 2 decreases from 346 to 6 (Table S1†). As the quasi-BIC mode generates high *Q* resonance with strong near-field enhancement, it satisfies the key requirement for sensing applications on the device surface.^21,27,29^

We again test another case with *D* = 360 nm with *H* varied from 0–100 nm, as shown in Fig. 3b. In this case, we observe a quasi-BIC mode 1 appearing at an etching depth of 10 nm. The *Q* factors of mode 1 and mode 2 also decrease with increasing *H* (Table S2†). Interestingly, the quasi-BIC resonance of mode 1 disappeared at *H* = 100 nm, where the Si is totally etched from the bottom. These results indicate that the variation of both, the silicon nanohole diameter and depth affect the BIC modes. Furthermore, the electric field of mode 1 displays patterns like vortices and antivortices from the top view and also show a great enhancement at *H* = 90 nm. According to the sectional and bottom drawings, we find that the electric field is enhanced at the interface between silicon and sapphire in both cases. The intense optical field at the surface and the intrinsic loss-free operation implies a strong interaction at the PhC slab that can lead to an extremely sensitive response to external environment perturbations.^21^ These results suggest the possibility of strong light–matter interaction for sensing applications.

**Fig. 4:**
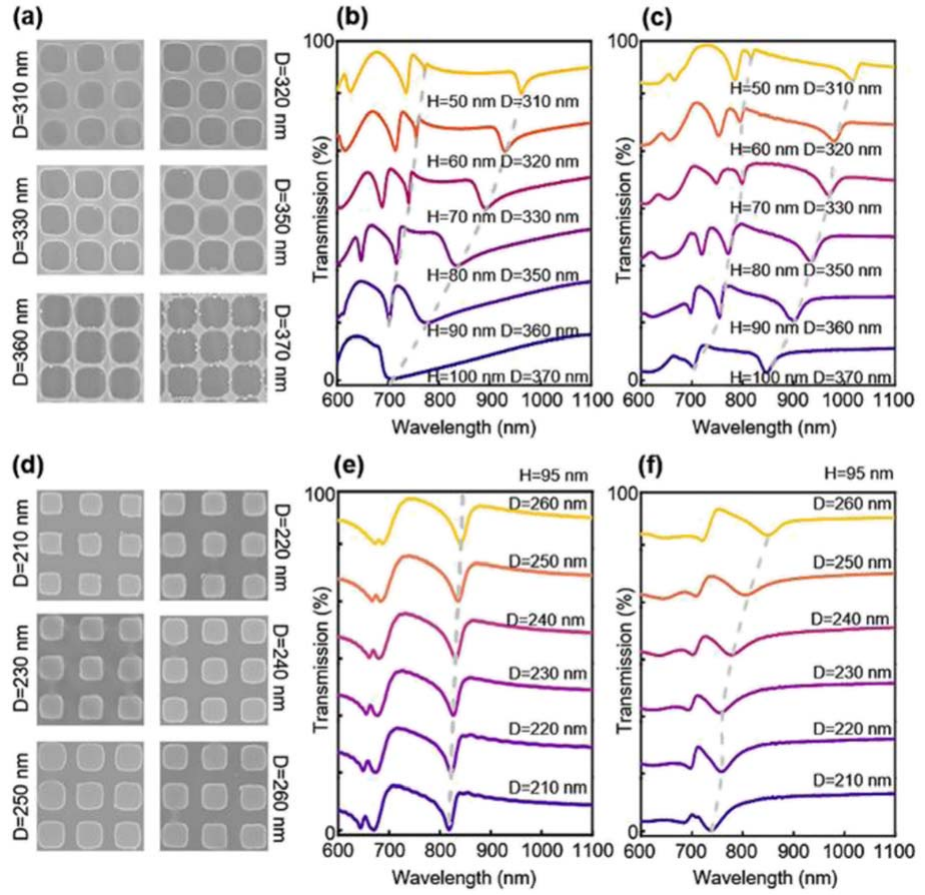
The simulated and experimental measured spectra change of different Si PhC structures. (a) and (d) Correspond to the scanning electron microscope images of nanoholes and nanopillars with different diameters, respectively. Simulated transmission spectra of nanoholes (b) and nanopillars (e) with different etching depths and diameters. Experimental transmission spectra of nanoholes (c) and nanopillars (f) structures with varying diameters.

As it reveales that the etching depth of silicon nanoholes can affect the quasi-BIC mode and *Q* factor, we have used SNL and RIE techniques to obtain etched nanohole PhC with different diameters and depths. In the oxygen etching step, we can easily control the diameter of the nanoholes by different etching times, and this time, instead of a long etch through time for the silicon layer, we use a shorter silicon etching time (140 s) to ensure that there is a thin layer of the silicon material (∼10 nm) left at the nanohole bottom. The SEM results corresponding to 140 s and 200 s silicon etching times are presented in Fig. S1,† where we can clearly observe a residue thin layer of the silicon film on the nanohole surface for the 140 s silicon etching, while a longer etching time of 200 s can completely yield a clean and smooth etch-through surface. The concave part or the height contrast of the photoresist is formed during the NIL process, and both the protruding and concave parts are etched at the same rate. Since the concave part is much thinner, they are etched (opened) very quickly, and the remaining protruding parts protect the underlying silicon from etching. Nonetheless, a longer etching time can erode the protruding part for lateral dimension tuning. The vertical dimension can also be easily controlled by the silicon etching time. Moreover, as discussed before,^30^ the silicon etching rate depends on the mask-opening size. A larger opening means a faster gas transport rate and faster etching, thus under the same etching conditions and time, a larger opening will result in a deeper etched depth. In our experiment, with the same silicon etching time, the nanohole PhC with a larger diameter *D* will have a deep etching depth of *H*. We can roughly estimate that the *H* obtained in the experiments is quite similar to the simulated values and varies from 50–100 nm. We compare our experimental measured spectra with FDTD simulation, and confirmed that we have made a series nanohole PhC with identical *D* and *H* (Fig. 4b and c). For example, the experimental and simulated spectra of *D* = 360 nm and *H* = 90 nm are almost identical with the *Q* factor of ∼90, and this structure is selected in all our sensing experiments.

As shown in Fig. 4b, c, e and f, the experimental results show an agreement with simulations. For the spectrum of nanoholes, mode 1 and mode 2 resonances show an obvious blueshift phenomenon with the increasing nanohole diameter, while redshift happens in nanopillars. The *Q* factor tends to stay stable with the changes in diameter in both cases (Fig. 4c and f, Tables 1 and S3†). Moreover, it is noticed that the PhC structure characterized using the scanning electron microscope is not perfectly shaped and exhibits many defects, which would broaden the linewidth of the resonant modes, leading to a low *Q* factor (Fig. 4a and d). Differently, the *Q* factor of mode 2 increases around one order of magnitude as the nanohole diameter changes (Fig. 4c, Table 1). In this situation, destructive interference occurs due to the vanishing of the coupling to the outside radiating channels, as a result, the nanohole diameter has a strong impact on the *Q* factor. At the singular diameter *D*_opt_, the *Q* factor is infinite but still maintains a high value for radii around it.^17^

**Table 1:** The resonance positions and calculated *Q* values of nanoholes (top) and nanopillars (bottom) with different diameters

Diameter	Resonant peak (nm)		Quality factor	
	Mode 1	Mode 2	Mode 1	Mode 2
310 nm	816	1017	136	59.8
320 nm	794	980	99.3	46.7
330 nm	799	969	99.8	51
350 nm	772	934	64.3	39
360 nm	754	900	83.7	37.5
370 nm	—	848	—	49.8
260 nm	849		24.9	
250 nm	801		20.5	
240 nm	777		20.4	
230 nm	755		22.9	
220 nm	758		23.7	
210 nm	737		29.5	

**Fig. 5:**
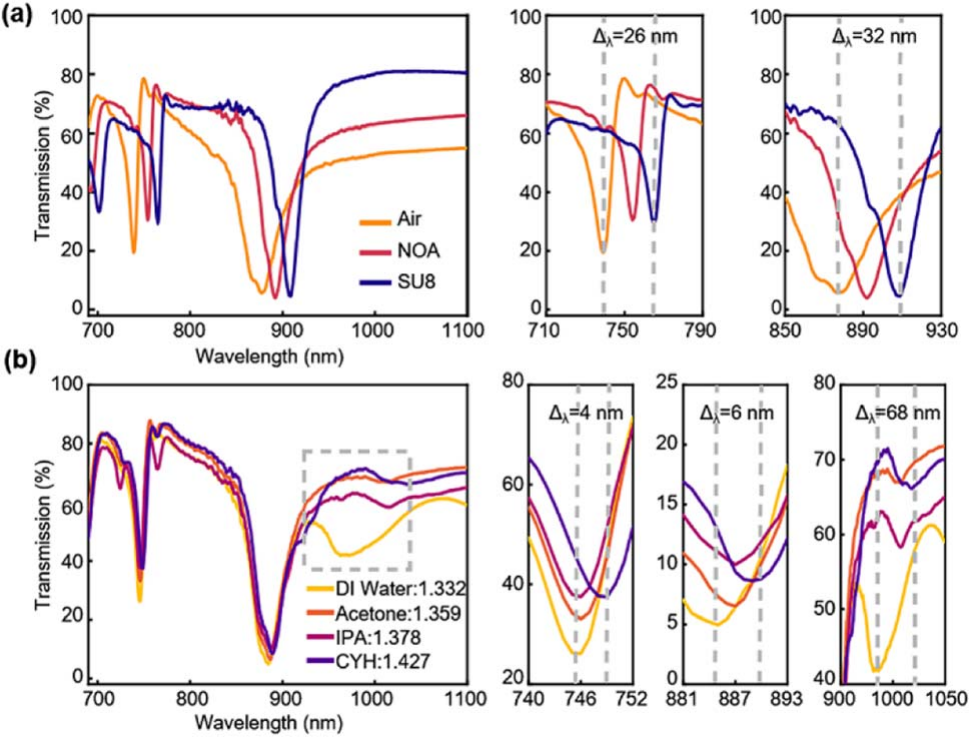
Bulk refractive index sensor sensitivity in different external environment. Transmission spectra are shown for (a) spin-coated NOA63 and SU8 devices, and (b) immersed in deionized water, acetone, isopropanol, and cyclohexane solutions.

To simplify the discussion, we use nanoholes with a reasonable good *Q* factor as an illustration to design sensing experiments. We cover the Si PhC slab with transparent dielectrics or immerse it in solutions with varying refractive indices, to investigate their performance as optical sensors. In the first case, two photoresists (SU8, *n* = 1.56 and NOA63, *n* = 1.6) have been spin-coated on the PhC surface and transmission spectra are measured using a UV-vis-NIR spectrophotometer, UV-3600, at room temperature. Compared with the spectrum in the air, both mode 1 and mode 2 in photoresists show an obvious redshift phenomenon (Δ*λ*_1SU8_ = 26 nm, Δ*λ*_2SU8_ = 32 nm; Δ*λ*_1NOA_ = 15 nm, Δ*λ*_2NOA_ = 15 nm), and the *Q* factors of mode 1 increase up to nearly twice due to the redshift phenomenon and decrease the resonance linewidth (Fig. 5a, Table 2). This indicates a sensitive response. Then we immerse the device in deionized water (DI water, *n* = 1.332), acetone (*n* = 1.359), isopropanol (IPA, *n* = 1.378), and cyclohexane (CYH, *n* = 1.427) to characterize the refractive index sensitivity of the sensor (Fig. 5b and S2†). As we expected, all the peaks shift to a longer wavelength direction as the refractive index increases and the results of experiments and simulations are consistent (Fig. S2 and S3†). FOM of mode 1 and mode 2 is around 1.66–3.36, the *Q* factor in the solution is similar to the *Q* factor obtained in the air (Table 2).

**Table 2:** Resonance position, calculated *Q* value, sensitivity and FOM corresponding to different refractive index substances

*n*	Resonant peak (nm)			Quality factor		Sensitivity (nm per RIU)			Figure of merit		
	Mode 1	Mode 2	Mode 3	Mode 1	Mode 2	Mode 1	Mode 2	Mode 3	Mode 1	Mode 2	Mode 3
Air: 1	739	877		92.4	33	—	—	—	—	—	—
NOA: 1.56	754	892		150.8	45	27	27				
SU8: 1.6	765	909		153	56.8	43.5	53.5				
DI water: 1.332	745	884	968	106	38.4	—	—	—	—	—	—
Acetone: 1.359	746	886	1014	93.2	34	37	74	1703	3.36	2.05	65.5
IPA: 1.378	746	887	1015	106.5	30.6	22	65.2	1022	2.2	1.59	32
CYH: 1.427	749	890	1036	107	33	42	63	716	3.5	1.66	10.2

Furthermore, it is a remarkable fact that the new mode (mode 3) with a broad linewidth shift Δ*λ* = 68 nm appears for cyclohexane measurement, which is the largest one compared to the other modes (mode 1: Δ*λ* = 4 nm, mode 2: Δ*λ* = 6 nm). We name it the higher-order or hybrid mode, which can be considered as a hybrid resonance mode between optical resonance and aqueous solution molecular absorption characteristics.^27,33^ A similar phenomenon has also been discovered and found recently in the crescent metasurface sensor,^27^ as higher-order resonance is commonly present in the aqueous solutions with an increased refractive index than air. From the results of our experiments, the high-order mode presents the highest sensitivity of 1703 nm per RIU, two orders of magnitude larger than mode 1 and mode 2, and the maximum FOM reached 65.5 in this case. Such a sensitive response is comparable to or even better than that of similar types of sensors,^21,27^ indicating an enormous potential to fabricate Si PhC for chemical and biological detection. We also want to highlight here, although this new mode is not quasi-BIC resonance, as we intend to form, its appearance may enlighten us to find more sensing modes under different application scenarios, as the optical resonance can couple to different surface refractive index changes to form new resonance modes.

**Fig. 6:**
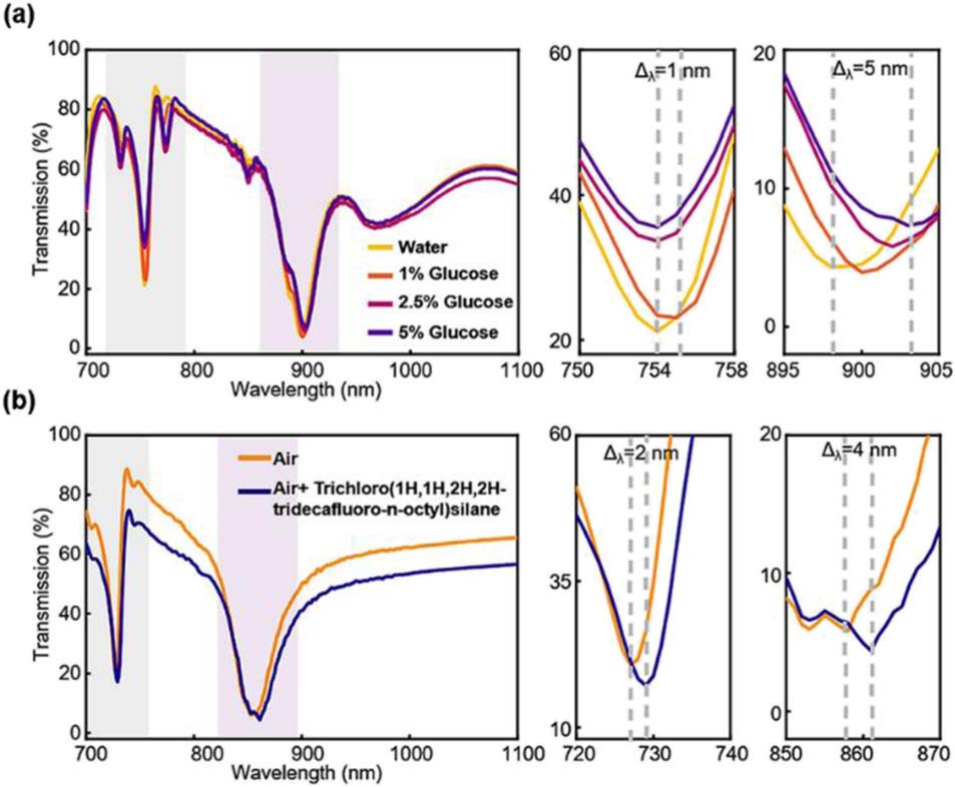
More sensor application demonstrations. (a) Transmission spectra of glucose solutions with different concentrations. (b)Transmission spectrum of the self-assembled monolayer (SAM) formed by trichloro(1*H*,1*H*,2*H*,2*H*-tridecafluoro-*n*-octyl)silane adsorbed on the sensor surface.

To evaluate the sensitivity of sensors approaching living situations, we have also designed a preliminary experiment for detecting glucose concentrations and molecular monolayers. Glucose solutions with concentrations of 1%, 2.5%, and 5% are used (Fig. 6a). The mode 2 resonance of 5% glucose shifts from 898 nm to 903 nm with a 5 nm step while mode 1 only moves less than 1 nm. When the solution is diluted to 1%, mode 2 resonance shifts 1 nm towards a longer wavelength compared to that of pure DI water. The results in Fig. 5b and 6a indicate that mode 2 is more sensitive to the refractive index changes at a longer wavelength. The results for distinguishing different concentrations indicate that mode 2 is the key sensing mode while mode 1 is not informative. Finally, the self-assembled monolayers (SAM) of trichloro(1*H*,1*H*,2*H*,2*H*-tridecafluoro-*n*-octyl)silane formed on the Si PhC surface (Fig. 6b) are studied. We measured apparent resonance shifts of 2 nm and 4 nm for mode 1 and mode 2 resonances, which are caused by the surface adsorption of the monolayer of molecules with ∼1 nm thickness. Particularly, this sensor achieves the optical detection of silane without any surface treatment and reaches the same level of response in the general approach.^34,35^

## Conclusions

We have investigated accidental BIC modes in large-area silicon PhC (nanoholes and nanopillars) formed by combined SNL and RIE techniques. Simulations demonstrate that the accidental BIC exists in an unpenetrated etch structure, where the bottom of nanoholes is covered with a thin silicon film. More importantly, the lateral and vertical dimensional changes of silicon PhC have been verified to cause the quasi-BIC mode appearance and tune the resonance position, spectrum shape, and *Q* factor. The mode position blueshifts as the nanohole diameter and depth increases. Moreover, the changing etching depth would impact the actual resonating modes, for instance, the appearance of mode 1 resonance at *H* = 42 nm suggestes a transformation from a true BIC mode to a quasi-BIC mode, which can be understood through the electric field intensity evolution. Experiments show similar results as the radius and height are varied together to yield different nanohole diameters and etching depths, and thus different resonating characteristics. Refractive index sensing shows the best sensitivity of 1703 nm per RIU and FOM of 65.5. Finally, we observe a good spectral shift for the solution concentration variations and monolayer silane molecule adsorption on the surface. Our high-throughput, low-cost sensing device might enable real large-scale optical sensors for various diagnostic applications.

## Experimental section

### Materials

Isopropyl alcohol (C_3_H_8_O; 99.7%, Shanghai Chemical Reagent, China), acetone (CH_3_COCH_3_; 99.5%, Nanjing Chemical Reagent, China), and cyclohexane (C_6_H_12_; 99.5%, Nanjing Chemical Reagent, China) were purchased to characterize the refractive index sensitivity (RIS) of the sensor. Glucose (C_6_H_12_O_6_; Sigma-Aldrich, America) and trichloro(1*H*,1*H*,2*H*,2*H*-tridecafluoro-*n*-octyl)silane (C_8_H_4_Cl_3_F_13_Si; 98%, Shanghai Haohong Biomedicine, China) were used to realize the sensing detection of the device.

### Device fabrication

A 100 nm-thick Si layer was first deposited on a sapphire substrate by plasma-enhanced chemical vapor deposition. After that, a layer of SU8 photoresist was spin-coated at a speed of 3000 rpm. The sample was heated on a 65° hot stage for 60 s, followed by a 95° heat stage for 60 s. The sample was then removed and cooled. During the imprinting process, the force was uniform to ensure that the structure was fully replicated. For the RIE (Tailong Electronics RIE) process, by adjusting the etching time, gas flow, and power parameters, a micro–nano structure with a similar size to the master was obtained. The first step was to etch a certain thickness of SU8 with an O_2_ gas plasma (gas flow rate 50 sccm, power 30 W). The second step used the pattern formed by the remaining SU8 photoresist as a mask, and the Si substrate was dry etched in a plasma etcher that resorted to a mix of CHF_3_ and SF_6_ gases for 140 s (gas flow rate 100/10 sccm, power 100 W) so that the silicon layer was not etched thoroughly. In the last step, the excess SU8 photoresist mask layer was removed with O_2_ gas (gas flow rate 50 sccm, power 100 W), and the cleaning time was 180 s to ensure that the excess photoresist was completely removed.

### Sensing measurement

In the experiment, we selected two photoresists with different refractive indices, NOA and SU8. In order to reduce the experimental error, we divided the sample from the same large area device into small parts. The NOA photoresist was irradiated with an ultraviolet lamp for 50 minutes to solidify on the sample surface. In the experiment, SU8 was applied to the sample surface using the spin coating instrument at 3000 rpm for 30 s. We used a UV-3600 UV-vis-NIR spectrophotometer to test the transmission spectrum of the sample, and the spot size was two millimetres. When testing the solvents with different refractive indices, we fixed the samples in the cuvette, and then added deionized water, acetone, isopropanol, and cyclohexane. After the test in one solvent, the sample was taken out and washed with ethanol until both the sample and cuvette were clean. The sample's transmission spectrum was measured to make sure the resonance was identical to that under initial conditions and the sensor measurement was repeated for different solvents. The operation steps of the glucose solution experiment were the same as above.

### Simulations

Simulations of the transmission spectra were performed using a simulation tool based on the FDTD method (FDTD Solutions, Lumerical, Canada). Simulations and experiments were performed with an unpolarized plane wave under normal incidence. A periodic boundary condition was applied along the *X* and *Y* directions, while a perfectly matched layer was set along the *Z* direction. The maximum mesh step was set as 3 nm.

### Morphologic and optical characterization

The scanning electron microscope (SEM) images of the fabricated samples were taken using a high-resolution SEM (S-4800, Hitachi). The optical transmission was measured under the normal incidence using a UV-3600 UV-vis-NIR spectrophotometer (Shimadzu, Japan).

## Conflicts of interest

The authors declare no conflicts of interest.

## Supplementary Material

NA-005-D3NA00001J-s001
