# Supplementary material for: Large-area silicon photonic crystal supporting bound states in the continuum and optical sensing formed by nanoimprint lithography
Source: Nanoscale Adv. 2023 Jan 25;5(5):1291–8. doi: 10.1039/d3na00001j (PMC9972860; doi:10.1039/d3na00001j)
Supplement: NA-005-D3NA00001J-s001 [file NA-005-D3NA00001J-s001.pdf]

Electronic Supplementary Material (ESI) for Nanoscale Advances.
This journal is © The Royal Society of Chemistry 2023

Supplementary information for

Large-Area Silicon Photonic Crystal Supporting Bound States in the Continuum and Optical Sensing Formed by Nanoimprint Lithography

Huijuan Zhao [†,a], Xinyi Cao [†,a], Qiao Dong[†,a], Chunyuan Song [a], Lianhui Wang[*,a], and Li Gao[*,a]

Contents of Supplemental Files:

Table S1 Quality factor of nanoholes with 300nm diameter while etching depth increase from 10nm to 100nm.

Table S2 Quality factor of nanoholes with 360nm diameter while etching depth increase from 10nm to 100nm.

Table S3 The key parameters of PhC are compared with those previously reported.

Figure S1 SEM characterization and comparison of Si PhC structure with insufficient and sufficient silicon etching time.

Figure S2 Supplementary sensitivity of sensor in different external environment.

Figure S3 Simulated Sensitivity of sensor in different external environment.

References

D =300nm				
Hight(nm)	Resonant Peak(nm)		Quality factor	
	Mode1	Mode2	Mode1	Mode2
10	-	1030	-	345.66
20	-	1023	-	255.75
30	-	1007	-	167.833
40	-	998	-	123.5
50	777	966	259	80.5
60	767	941	255	44.8095
70	759	913	253	24.6757
80	753	881	188	12.9559
90	746	847	124.333	8.55556
100	738	812	82	6.29457

Table S1 Quality factor of nanoholes with 300nm diameter while etching depth increasing from 10nm to 100nm.

D =360nm				
Hight(nm)	Resonant Peak(nm)		Quality factor	
	Mode1	Mode2	Mode1	Mode2
10	812	1032	-	258
20	798	1014	266	253.5
30	782	992	156.41	198.4
40	771	977	96.37	162.833
50	746	938	104	117.25
60	729	905	74.6875	64.6429
70	716	868	76	34.72
80	708	825	70.8	17.1875
90	702	776	87.75	9.12941
100	-	705	-	7.665

.

Table S2 Quality factor of nanoholes with 360nm diameter while etching depth increasing from 10nm to 100nm.

Reference	Geometry	Substrate	Controllable parameter	Quality factor	BIC wavelength
[1]	Cylindrical holes	Silica	Cylindrical hole size	2000	780nm
[2]	Tilted ellipses	Glass	Tilting angle	144	840-870nm
[3]	Crescent shape	Silica	Opening angle θ	118.4	750-900nm
[4]	Asymmetric double rods	Silica	Dimension of rod	136	710-750nm
[4]	Tilted ellipses	Silica	The major and minor axes	151	690-730nm
[4]	Split rings	Silica	The width of the ring	109	670-710nm
The paper	Circular nanohole	Sapphire	Nanohole size	136	750-820nm

Table S3 The key parameters of PhC are compared with those previously reported.

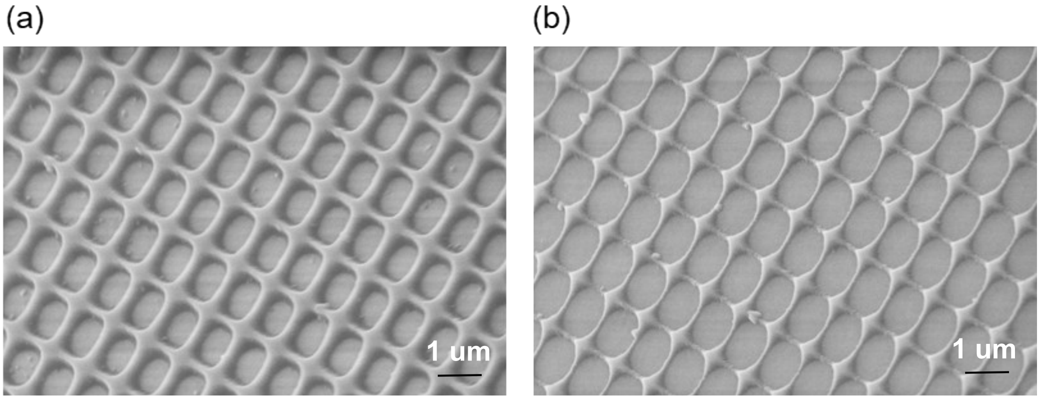

Fig. S1 SEM characterization and comparison of Si PhC structure with (a) insufficient silicon etching time of 140 s and (b) sufficient etching time of 200 s.

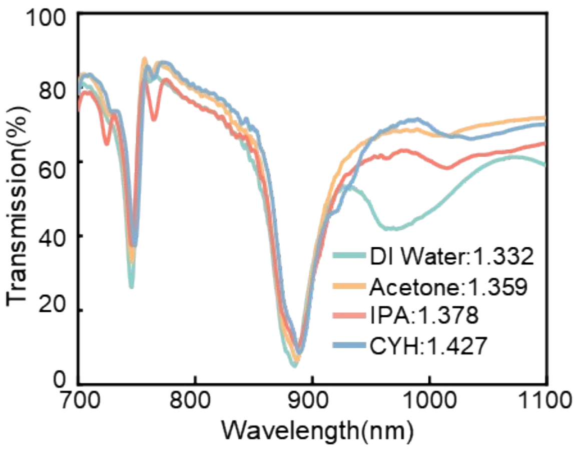

Fig. S2 A supplementary set of sensitivity of sensor immersed in deionized water, acetone, isopropanol, and cyclohexane solutions.

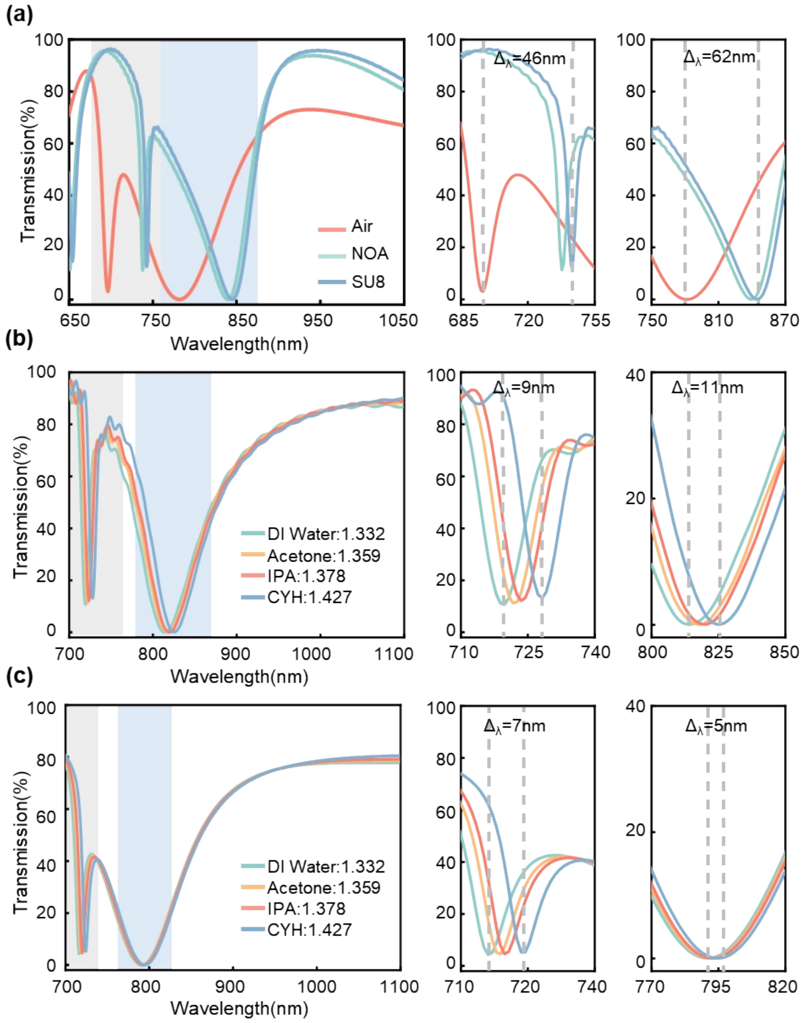

Fig. S3 Simulated Sensitivity of sensor in different external environment. Transmission spectra are shown in (a) spin-coated NOA63 and SU8 on metasurface. (b) Drop deionized water, acetone, isopropanol, and cyclohexane solutions onto the device. (c) Immersed in deionized water, acetone, isopropanol, and cyclohexane solutions.

References:

[1] S. Romano, G. Zito, S. Torino, G. Calafiore, E. Penzo, G. Coppola, S. Cabrini, I. Rendina and V. Mocella. *Photonics Res*. 2018. **6**, 726.

[2] F. Yesilkoy, E. R. Arvelo, Y. Jahani, M. Liu, A. Tittl, V. Cevher, Y. Kivshar and H. Altug. *Nat. Photonics*. 2019. **13**,390-396.

[3] J. Wang, J. Kühne, T. Karamanos, C. Rockstuhl, S. A. Maier and A. Tittl. *Adv. Funct. Mater*. 2021. **31**, 2104652.

[4]J. Kühne, J. Wang, T. Weber, L. Kühner, S. A. Maier and A. Tittl. *Nanophotonics*.2021. **10**, 4305-4312.
